# A spotlight on the tuberculosis epidemic in South Africa

**DOI:** 10.1038/s41467-024-45491-w

**Published:** 2024-02-12

**Keywords:** Tuberculosis

## Abstract

Tuberculosis is the leading cause of death from a single infectious agent, with over 25% of these occurring in the African region. Multi-drug resistant strains which do not respond to first-line antibiotics continue to emerge, putting at risk numerous public health strategies which aim to reduce incidence and mortality. Here, we speak with Professor Valerie Mizrahi, world-leading researcher and former director of the Institute of Infectious Disease and Molecular Medicine at the University of Cape Town, regarding the tuberculosis burden in South Africa. We discuss the challenges faced by researchers, the lessons that need to be learnt and current innovations to better understand the overall response required to accelerate progress.

**Figure:**
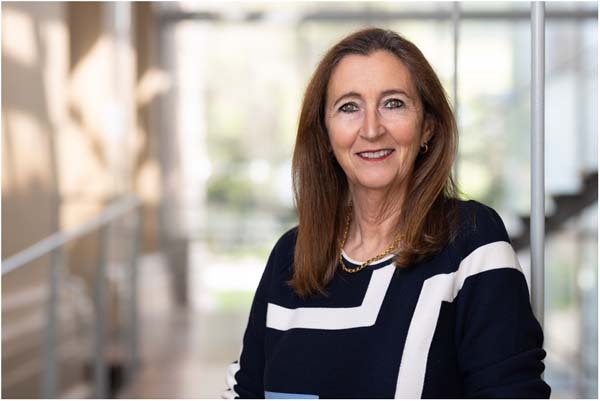
Professor Valerie Mizrahi

Given there is a vaccine against tuberculosis, the BCG, why does this disease remain such a problem in South Africa and what are the factors that contribute to this?

The evidence that infant BCG vaccination offers protection against tuberculosis (TB) in early childhood, especially against disseminated forms of the disease such as miliary TB or TB meningitis, is clear^1^. However, the effectiveness of infant BCG vaccination wanes over time, and, as a result, it offers little to no protection against pulmonary TB, the major form of the disease in adolescents and adults. Although South Africa has made significant strides in the fight against TB since 2010, as evidenced by the slow but steady decline in TB incidence and mortality, the incidence of TB in South Africa, estimated by the World Health Organisation to be 468 per 100,000 of the population in 2022^2^, remains stubbornly high for various reasons. South Africa is an upper middle-income country plagued by poverty, extreme income inequality and high levels of unemployment. As the prototypical disease of poverty, the major social determinants of TB in a high-burden country such as South Africa are undernutrition and overcrowding. Compounding these drivers are other biosocial risk factors; namely, HIV co-infection, alcohol use disorders, smoking, and diabetes. It is sobering to note that while South Africa runs the world’s largest HIV treatment program with 5.4 million people out of an estimated population of 60.4 million currently receiving antiretroviral therapy, no less than 54% of the estimated incident TB cases and 57% of the 54,000 deaths attributable to TB in 2022 occurred in HIV-infected persons^2^.

What aspects of this disease remain understudied and why is this?

There are major knowledge gaps throughout the infection-disease-transmission cycle of TB. Most immunocompetent persons infected with *Mycobacterium tuberculosis* cure or restrict the infection, and while significant progress has been made in understanding the mechanisms controlling later-stage infection, our understanding of early events in *M. tuberculosis* infection, and the mechanisms that can control infection at the earliest stages, and thereby restrict the tissue damage associated with TB, remains limited^3,4^. More generally, a deeper understanding of what cell types, cytokines, and molecular mechanisms are necessary and sufficient for protection is required as this would enable identification of specific biomarkers that define whether an infection was sterilized or controlled, that can predict whether an infection will progress to disease, that can monitor the response to treatment and establish whether cure was achieved, and are able to determine whether a vaccine will elicit a protective immune response.

The paucibacillary nature of TB, coupled with the microbiological complexities of *M. tuberculosis*, such as phenotypic heterogeneity^5^, differential culturability^6^ and persistence in the face of drug pressure and an effective immune response^7,8^ present myriad challenges for the microbiological investigation of TB in humans. At the most basic level, no test is currently available to determine accurately the number of viable tubercle bacilli in a human host. Enumerating *M. tuberculosis* in all the anatomical sites in which this organism might reside and understanding its various physiologic and replicative states in all the environments encountered during infection and disease in humans are very challenging problems^7^. What are those environments? Can they be modelled?^9^ It’s conceivable that some aspects of TB might not be amenable to microbiological investigation in humans, at least in the short to medium term. For the foreseeable future, we will therefore remain heavily reliant on the use of animal models of infection and disease, in particular nonhuman primates^10^, which mimic human disease most closely and have provided critical insights relevant to the pathogenesis of TB in humans^11,12^.

Another significantly understudied area of TB research, albeit one that appears poised for major advances in the future, is TB transmission^13^. To break chains of transmission, we need a far better understanding of the dynamics of TB transmission: Who is transmitting *M. tuberculosis*, and when, where and how does transmission to a new host occur? A major question in this regard is what the transmission potential of subclinical TB might be^14^. A recent TB prevalence survey conducted in South Africa revealed the scale of the problem of subclinical TB as evidenced by the fact that 58% of survey participants with TB disease reported no symptoms^15^. This finding is consistent with those from prevalence surveys in other countries and underlines the yawning detection gap created by the reliance on symptom screening to trigger TB testing and treatment. At a more fundamental level, we also need to understand the physiological adaptation of *M. tuberculosis* during transmission^16^. Which cellular function/s does *M. tuberculosis* engage in response to the multiple stresses imposed on the organism as it is expelled in the bioaerosol produced by an infected person, and suspended in the air before being inhaled by a new host? Could these functions be targeted to develop drugs that are highly effective at blocking transmission?

What are the biggest challenges that TB researchers are currently facing?

While TB researchers face myriad challenges^17^, the single greatest challenge, in my opinion, is the inadequate resourcing of the global TB research enterprise, which is acutely dependent on the vision, generosity and staying power of a handful of funding agencies and philanthropic organisations, most notably, the US National Institutes of Health and the Bill & Melinda Gates Foundation^18^. Decades of chronic underfunding have limited the scope of ambition of individual TB researchers and research consortia by curbing the ability to “dream big”. Resourcing impacts every facet of the TB research enterprise: the pace at which research projects can be executed; the ability to attract and retain the best and brightest talent from across the globe; and, critically, the ability to increase the TB science base by establishing and sustaining world-class laboratory and clinical research infrastructure and expertise in the places where they’re needed the most: research centres with biosafety level 3 facilities in high-burden countries. Being heavily (or even entirely) reliant on soft money, research entities within disease-endemic countries that conduct pivotal studies on TB operate in an ongoing state of precarity. While securing the core support needed to sustain the clinical research sites, expert staff and laboratory infrastructure is challenging, developing innovative ways to address this problem ought to be a top resourcing priority.

The resourcing gap reflects the woefully low prioritisation afforded to TB relative to other diseases. The magnitude of this problem was brought into sharp relief by the COVID-19 pandemic which demonstrated the level of funding that can in fact be mobilised to address a major global health challenge, when needed: US$100 billion was spent on COVID-19 research and development during the first eleven months of the pandemic. In contrast, an analysis of funding trends by the Treatment Action Group with support from the Stop TB Partnership revealed a total investment of $4.7 billion on TB research and development from 2018-2022—less than half the amount of $10 billion that world leaders had pledged at the United Nations High-Level Meeting on TB held in 2018^18^. It remains to be seen whether the resolution by the General Assembly of the United Nations adopting the “Political declaration of the high-level meeting on the fight against tuberculosis” on October 5, 2023, which commits to “mobilize adequate, predictable and sustainable financing for tuberculosis research and innovation especially to high-burden countries towards reaching 5 billion United States dollars a year by 2027”, is backed up by the political will needed to achieve this ambitious goal. In this regard, it is essential for TB to establish or maintain high visibility within global and national research agendas and strategic plans for other high-priority infectious disease challenges, including pandemic prevention, preparedness and response^19^, and antimicrobial drug resistance^20^.

In terms of the pace of research, progress in the TB field is frustrating slow; this applies to both the production of new knowledge and its application. The long duration—and hence, high cost—of clinical studies in TB is linked in part to a paucity of tools, which, if available, would have a transformative impact on the field. These include better biomarkers to predict the sterilising effect and duration of new drug regimens, and immune correlates of vaccine-mediated protection. Addressing these deficiencies is a top research priority, and one with which TB scientists in South Africa are actively engaged.

What have been the biggest advances in TB research in the last decade? Can you highlight current research that scientists in South Africa are driving forward?

Replacement of the binary paradigm in which TB was historically categorised as either “latent” or “active”, with a new paradigm in which TB is viewed instead as a spectrum or continuum of distinct disease states from latent to incipient, subclinical and active TB, stands out as one of the most important scientific advances in recent years^21,22^. This paradigm shift has profound implications for TB control by underscoring the urgent need for research to establish improved strategies to find, diagnose and treat people with TB at all points along the continuum, but especially those with early TB, before irreversible lung damage is sustained. In this context, it will be critically important to establish how shifting from the traditional, ‘one-size-fits-all’ strategy for the diagnosis and treatment of TB towards a more diversified or stratified approach would impact individual health and population health as well as health systems.

In another significant advance, persons with active TB were shown to aerosolise *M. tuberculosis* by tidal breathing^23^. Using a bespoke device known as the “Respiratory Aerosol Sampling Chamber”, scientists from the Desmond Tutu Health Foundation and the University of Cape Town captured the bioaerosol produced by TB patients and enumerated bacilli within the bioaerosol by live-cell fluorescence imaging using a solvatochromic trehalose probe, DMN-Tre^24^, which is incorporated enzymatically into the mycomembrane of metabolically active Actinobacteria, during separate respiratory manoeuvres: tidal breathing, forced vital capacity (FVC) and cough. The headline finding from this landmark study was that most bacilli aerosolised by symptomatic TB patients are contributed by tidal breathing rather than by cough – the signature symptom of TB. If one assumes that the number of live tubercle bacilli identified in patient bioaerosol correlates with patient infectiousness – a reasonable albeit untested assumption – then tidal breathing could be a dominant contributor to the transmission of *M. tuberculosis* from active TB cases to new hosts. Intriguing results from a follow-on study have demonstrated that, at presentation, TB clinic attendees aerosolise *M. tuberculosis* organisms independent of TB diagnosis, as determined by sputum-GeneXpert. This discovery suggests that unidentified individuals who aerosolise *M. tuberculosis* could contribute significantly to community exposure to the organism^25^.

In terms of translation, the development of new drugs and drug regimens which have revolutionised the treatment of drug-resistant TB, and the demonstration that treatment-shortening for drug-susceptible TB from six months down to four is achievable^26^, stand out as major advances which have signalled the start of an exciting new era in TB chemotherapy. An early study conducted in South Africa, and enabled by a clinical access program, demonstrated the profound impact of adding bedaquiline to an optimised background regimen on treatment outcomes of a cohort of pre-extensively drug resistant (XDR) and XDR-TB patients^27^. Subsequent trials to which South African researchers contributed significantly led the WHO to recommend bedaquiline-pretomanid-linezolid-moxifloxacin (BPaLM) as a six-month regimen for the treatment of rifampicin-resistant (RR) or multidrug resistant (MDR) TB^28^.

In the area of TB prevention, we’ve seen some positive signals from vaccine studies in the past few years, which have energised and brought a renewed sense of hope to the field of TB vaccinology. A Phase 2b trial of the M72/ASO1_E_ vaccine for prevention of progression from infection to disease in interferon gamma release assay (IGRA)-positive, HIV-uninfected adults demonstrated a vaccine efficacy of 49.7% at 3 years^29^ and inspired the recently announced Phase 3 trial of this vaccine for which $550 m in funding has been secured from the Bill & Melinda Gates Foundation and Wellcome Trust. In another encouraging development, a Phase 2 trial of BCG revaccination in IGRA-negative adolescents in a high-transmission setting demonstrated a reduction in the rate of sustained QuantiFERON-TB Gold In-tube assay (QFT) conversion with an efficacy of 45.4%^30^, which has led to a larger multicentre study in South Africa to assess whether a second dose of BCG given at 10 and 18 years of age, can help protect against TB. Importantly, samples collected from the two Phase 2b trials described above have provided a key resource for discovering immune correlates of protection against TB. Studies in this regard are being pursued through international collaborative research programs that involve large teams of leading TB immunologists, including those from the South African Tuberculosis Vaccine Initiative at the University of Cape Town, who are bringing state-of-the-art technologies to bear on this critically important problem^31^. In another key advance, intravenous administration of BCG was shown to prevent TB in rhesus macaques^32^ with subsequent work pointing to the early innate transcriptional response in peripheral blood as a potentially promising correlate of protection against TB^33^.

Over the past decade, there has also been spectacular progress on understanding the impact of genetic variation, whether natural or engineered, on the biology of *M. tuberculosis*. For example, genomic analyses of collections of clinical isolates from across the world are paving the way towards implementing sequencing-based resistance prediction in the clinic in the not-too-distant future^34^ and have uncovered the molecular basis of an “antibiotic resilience” phenotype exhibited by *M. tuberculosis* strains that carry mutations in a regulator, ResR, which enable faster resumption of growth after drug exposure^35^. In an exciting new discovery, the tight, PcaA-dependent packing of tubercle bacilli growing in cords was shown to protect *M. tuberculosis* against antibiotic-mediated clearance by allowing the bacilli to remain transcriptionally active under drug exposure and to re-grow after drug removal^8^. High-throughput functional genomic analyses by transposon mutagenesis and inducible CRISPR interference have enabled mycobacterial gene function to be probed at scale^36^. This approach has provided a new metric—the vulnerability index—for comparing, selecting and prioritising new TB drug targets^37^.

How can we better translate scientific findings from bench to the patient?

We need a multi-pronged approach to bridge gaps, which threaten the ability to translate findings rapidly and effectively from the bench to bedside and from there, to the community. On the one hand, the TB field urgently requires greater industry (both pharma and biotech) involvement; therefore, incentives are required to ensure that developers of new tools, be they drugs, vaccines, biomarkers or diagnostics, are not hamstrung by financial risk. The TB Drug Accelerator, a multi-sectoral, multi-party, multidisciplinary collaboration provides a useful public-private-academic partnership model which has demonstrated what can be achieved by creating a virtual collaborative network which, in this case, is focused on accelerating the development of new drugs and treatment-shortening regimens^38^. One the other hand, I feel strongly that we need more forums which bring together those working on the fundamental biology of TB and discovery of new tools, with healthcare workers on the frontlines of TB control to meet, exchange ideas, and learn from one another. Bridging the gap between the intellectual spheres in which scientists operate will be important to facilitate research that is targeted at, and effective in, translation. As we grapple with some of the dogma-challenging findings in *M. tuberculosis* biology and disease pathogenesis such as those highlighted above, having opportunities to consider their consequences from different perspectives will increase our ability to translate those discoveries into impactful interventions.

What are you currently most hopeful about?

As a “glass half full”-type optimist (a useful attribute for one working in this field!), I’m hopeful that safe and tolerable drug regimens that effect durable cure in two months or less will be developed within the next decade. I am also convinced that much greater attention will be paid to the detection and treatment of subclinical TB, and that this will have a major impact on the epidemic. I expect to see insights emerging from research at the nexus of human TB immunology and vaccinology, which will impact significantly on prevention research. Perhaps most importantly, I am inspired by the talent, passion and commitment of TB scientists with whom I work, and from whom I learn—especially the next generation of researchers. This makes me confident that scientists based in high-burden countries such as mine are poised to play an increasingly prominent role in setting the global agenda for TB research, and in the discovery, development, and implementation of new tools for controlling this age-old disease.
